# Endosonographic Features of Histologically Proven Gastric Ectopic Pancreas

**DOI:** 10.1155/2014/160601

**Published:** 2014-10-12

**Authors:** Jen-Wei Chou, Ken-Sheng Cheng, Chun-Fu Ting, Chun-Lung Feng, Yu-Ta Lin, Wen-Hsin Huang

**Affiliations:** ^1^School of Medicine, China Medical University, Taichung 40447, Taiwan; ^2^Division of Gastroenterology and Hepatology, Department of Internal Medicine, China Medical University Hospital, No. 2, Yude Road, North District, Taichung 40447, Taiwan

## Abstract

Gastric ectopic pancreas is an uncommon developmental anomaly and its histological diagnosis is usually difficult by using a conventional biopsy forceps. In the literature, most cases of gastric ectopic pancreas were usually diagnosed by gross pattern during endoscopic examination or features of endoscopic ultrasound. In contrast, this disease was seldom diagnosed by histology in clinical practice. Although the typical endoscopic ultrasonographic features of ectopic pancreas include heterogeneous echogenicity, indistinct borders, and a location within 2 or more layers, it can also exhibit hypoechoic homogeneous echogenicity and a distinct border within the fourth sonographic layer (muscularis propria) similar to the endoscopic ultrasonographic features of gastrointestinal stromal tumors. In our study, we found that 53% of gastric ectopic pancreas originated within the fourth sonographic layer, demonstrating hypoechoic, homogeneous echogenicity, and distinct borders. Therefore, recognizing endoscopic ultrasonographic features, combining with deep biopsy, endoscopic ultrasound-guided fine needle aspiration/core needle biopsy can prevent conducting unnecessary resection. Surgical resection is the mainstay treatment for symptomatic gastric ectopic pancreas, but endoscopic resection using endoscopic mucosal resection or endoscopic submucosal dissection technique provides an alternative method of removing superficial-type and deep-type gastric ectopic pancreas.

## 1. Introduction

Ectopic pancreas, the presence of pancreatic tissue outside its typical location without anatomic or vascular connections to the pancreas, is a uncommon disease in clinical practice [1]. This uncommon developmental anomaly is also referred to as heterotopic or aberrant pancreas. The estimated incidence at autopsy ranges from 0.5% to 13% in the general population [2]. Ectopic pancreas is typically incidentally discovered during surgical explorations or endoscopic examinations of the gastrointestinal (GI) tract [3].

Ectopic pancreas is not typically diagnosed using histology because (1) conventional endoscopic biopsy forceps are limited when seeking deep specimens and (2) resection is uncommonly required in asymptomatic patients [4]. However, the differential diagnosis of ectopic pancreas from other subepithelial tumors, such as carcinoid tumors, lymphomas, or gastrointestinal stromal tumors (GISTs) is crucial [5]. Computed tomography and magnetic resonance imaging provide information for differential diagnosis of GI tumors, but they are limited for diagnosing small lesions within the gastric wall [6, 7]. In contrast, endoscopic ultrasonography (EUS) can assist in distinguishing these small subepithelial lesions. Although EUS has been shown to be a useful modality in diagnosing gastric ectopic pancreas in the literature, some reported cases were not histologically proven. In this study, we described the EUS features of histologically proven gastric ectopic pancreas.

## 2. Patients and Methods

From May 2006 to December 2013, we retrospectively reviewed patients who underwent EUS for a gastric subepithelial tumor at China Medical University Hospital, a tertiary referral hospital in the middle of Taiwan. All patients firstly underwent esophagogastroduodenoscopy (EGD) because of dyspepsia or epigastric pain. Thirteen patients with histologically proven gastric ectopic pancreas were included during the study period. Written informed consent was obtained from all patients.

EUS was performed using a radial echoendoscope at a scanning frequency of 7.5 or 12 MHz (Olympus GF-UM 240; Olympus, Tokyo, Japan), and an ultrasonic miniprobe at a scanning frequency of 12 MHz (Olympus UM-2R; Olympus, Tokyo, Japan) was introduced using an electronic esophagogastroduodenoscope (Olympus XQ-240; Olympus, Tokyo, Japan). All patients were administered intravenous sedatives during the examination. The normal gastric wall under EUS is usually visualized as a five-layered structure, which correlates with the histologic layers: the first hyperechoic layer on EUS correlates to the superficial mucosa, the second hypoechoic layer to the deep mucosa, the third hyperechoic layer to the submucosa, the fourth hypoechoic layer to the muscularis propria, and the fifth hyperechoic layer to the serosa and subserosal fat.

The EUS features of gastric ectopic pancreas were recorded, including the location, size, gross shape (Yamada classification [8]), central dimpling, sonographic layer of origin, echogenicity, homogeneity, presence of anechoic duct-like structures, and border. Moreover, these lesions were also divided based on the Park classification (superficial type originated in the second and/or third layers; deep type originated in the third and fourth layers with or without extension into the fifth layer) [4]. Pathological types were applied according to the Heinrich classification [9].

### 2.1. Statistical Analysis

The statistical significance of differences between the locations of ectopic pancreas was assessed by using the *χ* ^2^ test or Fisher's exact test. The size was assessed by using Student's *t*-test. A *P* value < 0.05 was considered statistically significant. Statistical calculations were performed with SPSS version 12.0 for Windows (SPSS Inc., Chicago, IL, USA).

## 3. Results

In the period of study, a total of 13 patients with histologically proven ectopic pancreas were enrolled. The clinicopathological characteristics in 13 patients with gastric ectopic pancreas were summarized in Table 1. These participants were 5 men (38%) and 8 women (62%), with a mean age of 40 years (range, 20–77 years). Among the lesions, 8 (8/13, 62%) were located in the antrum, 4 (4/13, 31%) were in the body, and one (1/13, 7%) was in the angulus. In the term of gross shape by EGD, 5 lesions demonstrated Yamada Type I and 8 were Yamada Type II. Central dimpling was noted in 7 lesions. According to the Heinrich pathological classification, 5 lesions were Type I and 8 were Type II. Ectopic pancreas was confirmed using a biopsy (*N* = 5), surgical wedge resection (*N* = 4), or endoscopic submucosal dissection (ESD) (*N* = 4).

**Figure 1:**
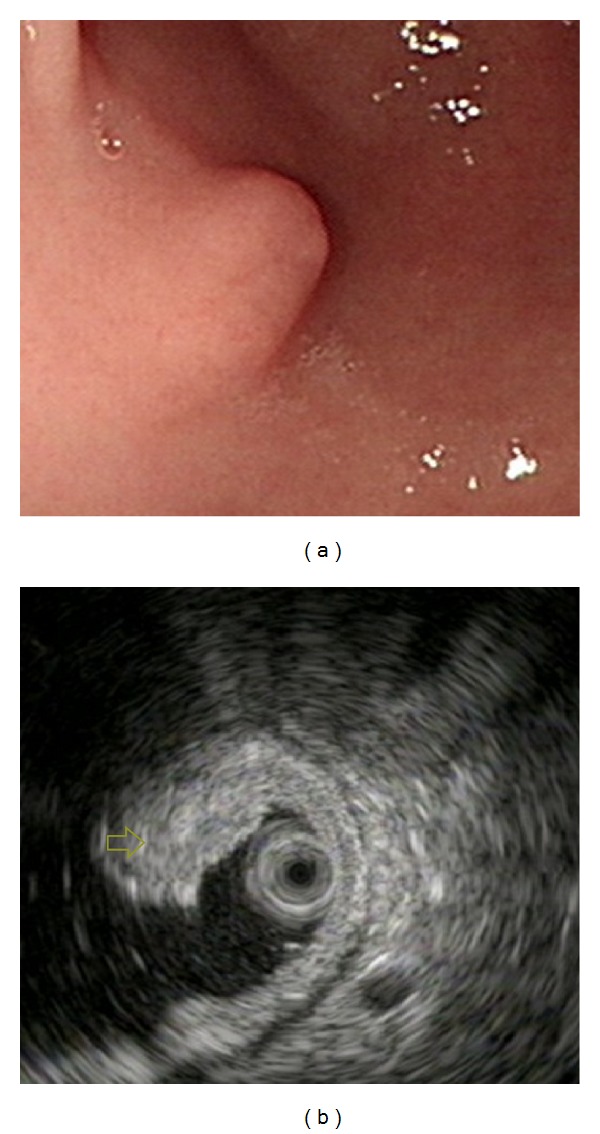
Ectopic pancreas originates from the second and third sonographic layers of the gastric wall. (a) A subepithelial tumor with intact but uneven surface was identified in the antrum. (b) Endoscopic ultrasonographic image obtained with a 12 MHz catheter probe. The tumor exhibits heterogeneous, mixed echoic echogenicity and indistinct margins, involving the second and third sonographic layers of the gastric wall (arrow).

**Figure 2:**
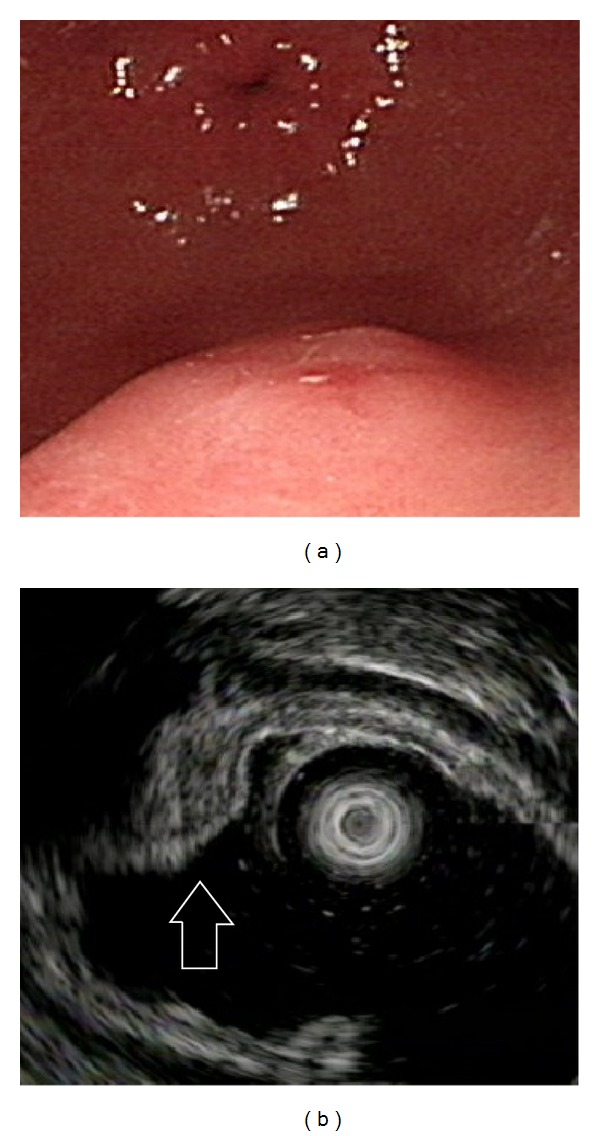
Ectopic pancreas originates from the second and third sonographic layers of the gastric wall. (a) A subepithelial tumor with intact but uneven surface was identified in the antrum. (b) Endoscopic ultrasonographic image obtained with a 12 MHz catheter probe. The tumor exhibits heterogeneous, mixed echoic echogenicity and indistinct margins, involving the second and third sonographic layers of the gastric wall (arrow).

**Figure 3:**
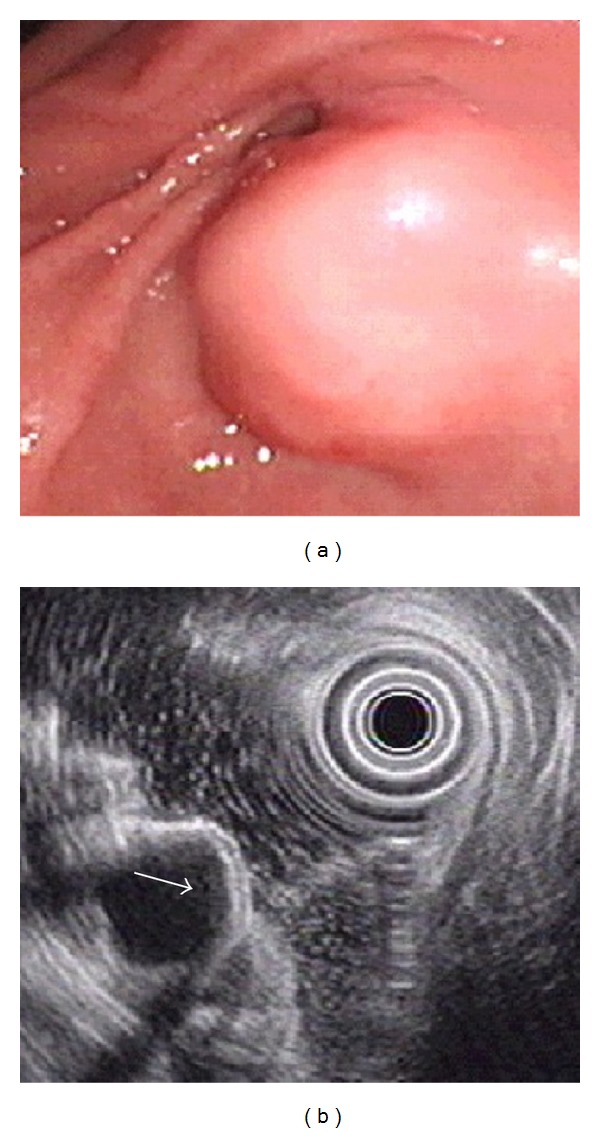
Ectopic pancreas originates from the fourth sonographic layers of the gastric wall. (a) A subepithelial tumor with intact mucosa was identified at antrum. (b) Endoscopic ultrasonographic image obtained with a 12 MHz catheter probe. The tumor exhibits homogenous, hypoechoic echogenicity and distinct margin originating from the fourth sonographic layer of gastric wall (arrow).

**Figure 4:**
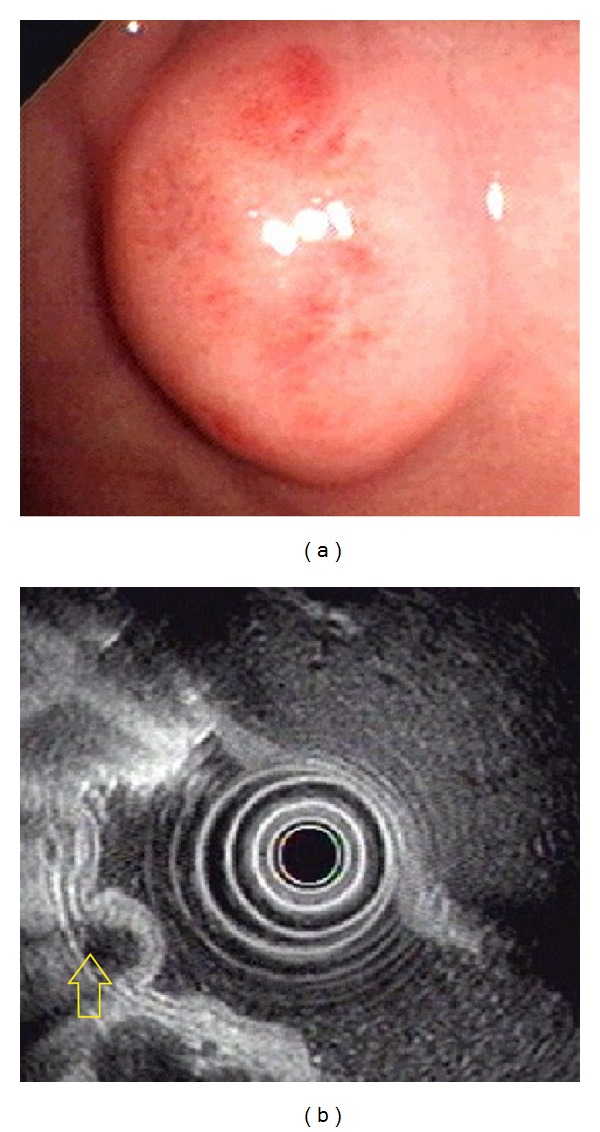
Ectopic pancreas originates from the fourth sonographic layers of the gastric wall. (a) A subepithelial tumor with intact mucosa was identified at antrum. (b) Endoscopic ultrasonographic image obtained with a 12 MHz catheter probe. The tumor exhibits homogenous, hypoechoic echogenicity and distinct margin originating from the fourth sonographic layer of gastric wall (arrow).

The features of EUS in patients with gastric ectopic pancreas were summarized in Table 2. The sizes of gastric ectopic pancreas ranged from 10 to 23 mm (mean 16 ± 5 mm). Nine lesions (69%) exhibited hypoechoic echogenicity and 4 (30%) exhibited mixed echoic echogenicity (Figures 1 and 2). Seven lesions (53%) were homogeneous (Figures 3 and 4). The borders were distinct in 10 lesions (76%) and indistinct in 3 lesions (24%). Anechoic tubular structures appeared in only one lesion (7.6%). Eleven lesions involved one layer of the gastric wall, 7 originated in the fourth layer, and 4 originated in the second layer; the remaining 2 lesions involved both the second and third layers. Seven lesions were the deep type (53%) and 6 were the superficial type (47%).

The EUS features of patients with gastric ectopic pancreas originating in the fourth sonographic layer or not were summarized in Table 3. When the ectopic pancreas was classified by the location, the lesions originated in the fourth layer showed a higher frequency of hypoechoic echogenicity (100.0% versus 16.6%, resp., *P* = 0.005) and homogeneous homogeneity (87.5% versus 0.0%, resp., *P* = 0.015) than those not in the fourth layer. In addition, the lesions in the fourth layer were more common to exhibit both hypoechoic and homogeneous features than those not in the fourth layer (85.7% versus 16.6%, resp., *P* = 0.029).

## 4. Discussion

Ectopic pancreas might exhibit any components of a normal pancreas, including acini, ducts, and islets of Langerhans [10]. In 1909, Heinrich proposed 3 types of ectopic pancreas in histology. Type I contains acini, ducts, and islets of Langerhans. Type II contains incomplete or lobular arrangement and lacks endocrine elements. Type III comprises ectopic tissue of proliferating ducts, exhibiting neither acini nor endocrine elements [9]. In our present study, 5 lesions (38.4%) were Heinrich Type I and 8 (61.5%) were Type II.

The common site of ectopic pancreas is located in the GI tract: stomach (26%–38%), duodenum (28%–36%), and jejunum (16%) [10]. Lesions have also been reported in the colon, spleen, liver, Meckel's diverticulum, gallbladder, bile ducts, or fallopian tubes [11]. The gross appearance of a typical ectopic pancreas in the stomach is a firm, round, or oval subepithelial lesion. Central dimpling (also called central umbilication) caused by the opening of a duct may also be observed. This implies that ectopic pancreas can be presumptively diagnosed before obtaining histologic results. In previous reports, central dimpling was observed in 34.6%–90.0% of patients with ectopic pancreas [4, 5, 12]. In our present study, cenreal dimpling was present in 53.8% of patients. Anechoic cystic (or tubular) structure, known as the component of pancreas duct, is another specific EUS feature for ectopic pancreas [13]. However, anechoic area in EUS was only present in 1 lesion of all patients with ectopic pancreas (7.6%) in our present study.

Matsushita et al. revealed useful EUS features for establishing a preoperative diagnosis of ectopic pancreas, namely, indistinct borders, heterogeneous echogenicity, the presence of an anechoic area, and a location within the second, third, and/or fourth layers [13]. Park et al. described other characteristic EUS features of ectopic pancreas, such as lobulated margins, a mural growth pattern, and localization within 2 or more layers [4]. However, in our present study, 7 of 13 lesions (53.8%) were hypoechoic and homogeneous. The EUS findings somewhat differ from some previous reports in the literature. This discrepancy might result from interobserver variation in interpreting the EUS images and is suspected because of the hypoechoic homogeneous characteristics of the fourth sonographic layer ectopic pancreas in EUS. In our previous study by Chen et al. in 2008, he also found a case of ectopic pancreas located within the fourth sonographic layer (muscularis propria) [5]. In our present study, the percentage of patients with ectopic pancreas exhibiting a fourth sonographic layer was larger compared with other studies. In our study, seven lesions (7/13, 53.8%) of ectopic gastric pancreas were located within the fourth sonographic layer, and six of these lesions (6/7, 85.7%) were hypoechoic and homogeneous. However, in previous reports by Matsushita et al. and Park et al., they did not find any patients with gastric ectopic pancreas located within the fourth layer only. In addition, according to the statistical analysis, the lesions in the fourth layer were more common to exhibit both hypoechoic and homogeneous feature than those not in the fourth layer (85.7% versus 16.6%, resp., *P* = 0.029). It suggests that ectopic pancreas within the fourth layer exhibited various EUS features. In our present study, the border was distinct in 10 lesions (76.9%). The 7 lesions in the fourth layer all exhibited a distinct border (100%). This might be because the border in a hypoechoic lesion in the muscularis propria layer of the stomach is relatively easy to distinguish from the hyperechoic area in the submucosa and serosa layers. Although the statistical analysis showed no significant differences between lesions in the muscularis propria layer and not in the muscularis propria layer (100.0% versus 50.0%, resp., *P* = 0.07) because of small number of our cases, our results differed from the reports of Matsushita et al. and Park et al., in which most ectopic pancreas exhibited indistinct borders. Despite our new EUS findings of gastric ectopic pancreas, a smooth lesion in antrum or duodenum, with a central dimple, with a tubular structure within it, with mixed echogenicity and involving the submucosa and the muscularis propria would have a pretty high chance of being an ectopic pancreas.

The clinical presentations of patients with ectopic pancreas are usually asymptomatic or nonspecific symptoms such as abdominal pain, nausea, and vomiting. However, some rare complications of ectopic pancreas have been reported, including perforation, GI bleeding, gastric outlet obstruction, obstructive jaundice, intestinal obstruction, and intussusceptions [9, 14]. Carcinoma developing in an ectopic pancreatic tissue was rarely reported in the literature [15, 16]. Thus, resection is usually unnecessary for asymptomatic patients. In our present study, all patients underwent EGD because of dyspepsia or epigastralgia. Gastric subepithelial tumors were discovered and EUS was performed later. Eight patients (61.5%) underwent tumor resection due to the preoperative diagnosis of gastric GISTs by EUS. Thus, resection can be implemented following a misdiagnosis. Most GISTs are hypoechoic and homogeneous lesions, which exhibit distinct margins and typically originate within the fourth layer of the GI tract [17]. In our present study, gastric ectopic pancreas in the fourth layer shared similar features as GISTs. Thus, in addition to GISTs, ectopic pancreas is a possible diagnosis of a hypoechoic heterogeneous lesion originating from the gastric fourth layer with distinct margins. Bite-on-bite biopsy, EUS-guided fine needle aspiration (EUS-FNA), and EUS-guided core needle biopsy are valuable modalities in evaluating these lesions [18]. Relevant pathological findings could prevent unnecessary resections.

Park et al. classified the ectopic pancreas into 2 types based on the sonographic layer of origin: the superficial type and deep type. If symptoms are noted owning to ectopic pancreas, the superficial type is an optimal candidate for endoscopic resection. However, the deep type might require a surgical approach [4]. In the past, endoscopic mucosal resection (EMR) in treating gastric ectopic pancreas has been reported in the literature [19, 20]. Recently, ESD is a newly developed method for removing subepithelial tumors of the GI tract [21, 22]. Ryu et al. performed ESD for 4 cases of gastric ectopic pancreas after failure of EMR [23]. Liu et al. also reported 9 patients who underwent ESD and EMR for symptomatic ectopic pancreas [14]. In our present study, ESD was performed for 2 patients who exhibited deep type (the muscularis propria layer) ectopic pancreas without complications. Thus, ESD appears to be relatively safe for the complete resection of small (≤20 mm) gastric subepithelial tumors, including ectopic pancreas, originating from the muscularis propria layer [24].

## 5. Conclusions

The typical EUS features of ectopic pancreas include heterogeneous echogenicity, indistinct borders, and a location within 2 or more layers. However, it can also exhibit hypoechoic homogeneous echogenicity and a distinct border within the muscularis propria layer similar to the EUS features of GISTs. Deep biopsy relevant EUS features by using EUS-guided FNA/core needle biopsy can make a differential diagnosis of gastric subepithelial tumors before resection. Surgical resection is the mainstay treatment for symptomatic gastric ectopic pancreas, but endoscopic resection using EMR or ESD technique provides an alternative method of removing superficial type and deep type gastric ectopic pancreas.

However, our present study involves certain limitations. First, the number of patients was small because pathological diagnosis is not always possible; thus, we could not exclude the possibility of selection bias. Second, because a pathological diagnosis was not made in all of the cases showing typical EUS findings for ectopic pancreas, a selection bias could exist.

## Figures and Tables

**Table 1:** Clinicopathological characteristics in 13 patients with gastric ectopic pancreas.

Case	Gender	Age	Symptom for EGD	Location	Gross shape∗	Dimpling	Method of pathological confirmation	Pathological type^†^
1	F	40	Dyspepsia	High body	II	Yes	Biopsy	II
2	M	36	Epigastric pain	Antrum	I	No	Biopsy	II
3	F	30	Epigastric pain	Antrum	II	No	Biopsy	I
4	F	20	Dyspepsia	Antrum	I	Yes	Biopsy	II
5	M	43	Epigastric pain	Middle body	I	Yes	Biopsy	II
6	M	30	Epigastric pain	Antrum	II	No	Laparotomy	II
7	F	77	Dyspepsia	Angulus	I	No	Laparotomy	I
8	M	39	Dyspepsia	Middle body	II	Yes	Laparotomy	I
9	F	28	Epigastric pain	Lower body	I	Yes	Laparoscopy	I
10	F	31	Dyspepsia	Antrum	II	No	ESD	I
11	F	51	Epigastric pain	Antrum	II	Yes	ESD	II
12	F	42	Dyspepsia	Antrum	II	Yes	ESD	II
13	M	64	Dyspepsia	Antrum	II	No	ESD	II

*By Yamada classification [8].

^†^By Heinrich classification [9].

EGD, esophagogastroduodenoscopy.

ESD, endoscopic submucosal dissection.

**Table 2:** Endoscopic ultrasonographic features in 13 patients with gastric ectopic pancreas.

Case	EUS features						
	Size (mm)	Layer	Echogenicity	Homogeneity	Anechoic area of duct	Border	EUS classification^#^
1	12	4	Hypoechoic	Homogenous	No	Distinct	D-type
2	10	2	Mixed echoic	Heterogeneous	No	Distinct	S-type
3	12	2, 3	Mixed echoic	Heterogeneous	No	Indistinct	S-type
4	11	2, 3	Mixed echoic	Heterogeneous	No	Distinct	S-type
5	12	2	Mixed echoic	Heterogeneous	No	Indistinct	S-type
6	20	4	Hypoechoic	Homogenous	No	Distinct	D-type
7	22	4	Hypoechoic	Homogenous	No	Distinct	D-type
8	23	4	Hypoechoic	Homogenous	Yes	Distinct	D-type
9	20	4	Hypoechoic	Homogenous	No	Distinct	D-type
10	10	2	Hypoechoic	Homogenous	No	Distinct	S-type
11	20	4	Hypoechoic	Heterogeneous	No	Distinct	D-type
12	20	4	Hypoechoic	Homogenous	No	Distinct	D-type
13	21	2	Mixed echoic	Heterogeneous	No	Indistinct	S-type

^#^By Park's classification [4].

**Table 3:** Endoscopic ultrasonographic features of 13 patients with gastric ectopic pancreas originating in the fourth sonographic layer or not.

	In the 4th layer	Not in the 4th layer	*P* value
Gross shape (by Yamada classification 11)			1.000
I	3	2	
II	4	4	
Dimpling/umbilication			0.286
Present	5	2	
Absent	2	4	
Size (mm, mean SD)	19.5 ± 2.1	12.6 ± 2.7	0.008
Echogenicity			0.005
Mixed echoic	0	5	
Hypoechoic	7	1	
Homogeneity			0.015
Homogeneous	6	0	
Heterogeneous	1	5	
Hypoechoic and homogeneous			0.029
Yes	6	1	
Not	1	5	
Border			0.070
Distinct	7	3	
Indistinct	0	3	
Anechoic duct-like structure			1.000
Present	1	0	
Absent	6	6	
